# Digestive Tract Structure and Seasonal Dynamics of Gut Microbiota in *Hypomesus nipponensis* from Bosten Lake

**DOI:** 10.3390/ani16111595

**Published:** 2026-05-23

**Authors:** Xinnan Fu, Qian Xiao, Wenjie Ma, Sitong Li, Zhelan Wang, Kai Deng, Junjie Zhang

**Affiliations:** 1 College of Life Sciences, Xinjiang Agricultural University, Urumqi 830052, China; 18087035850@163.com (X.F.); qianxiao181@163.com (Q.X.); 18160510177@163.com (S.L.); m15286133601@163.com (Z.W.); 2 Xinjiang Key Laboratory for Ecological Adaptation and Evolution of Extreme Environment Organisms, College of Life Sciences, Xinjiang Agricultural University, Urumqi 830052, China; 18712517561@163.com; 3 College of Modern Fishery Industry, Xinjiang Agricultural University, Urumqi 830052, China; 4 Xinjiang Characteristic Aquatic Research Center, Urumqi 830052, China; 5 Xinjiang Bosteng Lake Ecological Fisheries Co., Ltd., Bohu 841400, China; 18087895148@163.com

**Keywords:** *Hypomesus nipponensis*, gut microbiota, seasonal variation, digestive tract structure

## Abstract

Digestive tract structure is a key indicator of fish health and environmental adaptation, while seasonal dynamics of the gut microbiota reflect host responses to environmental changes. In this study, the digestive tract microstructure of *Hypomesus nipponensis* from Bosten Lake was characterized using H&E staining and scanning electron microscopy, followed by 16S rDNA gene V3-V4 region sequencing and analysis of the gut microbiota in spring, summer, and autumn. The results showed that the esophageal mucosa of *H. nipponensis* is a stratified columnar epithelium, with abundant gastric glands, and the circular muscle layer of the stomach caeca is significantly thickened (244.84 ± 49.01 μm). The pyloric caeca resemble the gut in structure; both are covered with dense microvilli on the luminal surface. Collectively, these features constitute the structural basis for its carnivorous diet. Microbiota analysis revealed that the diversity of gut microbiota fluctuated significantly with season: the Chao, Ace, and Sob indices in spring (144.63 ± 30.27) were significantly higher than in summer (82.13 ± 21.45) and autumn (83.25 ± 15.30) (*p* < 0.001), with no significant difference between summer and autumn (*p* > 0.05). The dominant marker genera of *H. nipponensis* in spring, summer, and autumn were *Bacillus* (31.60%), *Clostridium* (32.20%), and *Sarcina* (29.32%), respectively. This study describes the adaptive characteristics of the digestive tract structure and feeding habits of *H. nipponensis* and reveals the seasonal changes in its gut microbiota. Importantly, since the digestive tract structure data were collected only in summer, the direct relationship between the structure and seasonal microbial dynamics cannot be determined, and multi-season histological sampling is needed for further investigation. Nevertheless, these findings provide preliminary morphological and microbiological references for the ecological adaptation of this species in Bosten Lake and offer a scientific basis for water resource management in this area.

## Simple Summary

This study was conducted to provide an integrated description of the digestive tract structure of *Hypomesus nipponensis*, determined using histology and ultrastructure, and to analyze its gut microbiota in spring, summer, and autumn via 16S rDNA V3-V4 sequencing. The results showed that the digestive tract of *H. nipponensis* possesses abundant gastric glands, and both the pyloric caeca and the gut are covered with dense microvilli. Richness indices (Chao, Ace, and Sob) in spring (144.63 ± 30.27) were significantly higher than those in summer (82.13 ± 21.45) and autumn (83.25 ± 15.30); the dominant marker genera were *Bacillus* (31.60%) in spring, *Clostridium* (32.20%) in summer, and *Sarcina* (29.32%) in autumn. These results indicate that the specialized digestive tract structure provides stable histological support for feeding, and the seasonal succession of gut microbiota responds to environmental fluctuations in Bosten Lake.

## 1. Introduction

Digestive tract morphology and function reflect the adaptive evolution of fish to complex habitats [1,2,3]. Differences in digestive tract structure exist among fish with different dietary habits [4,5,6]. Typically, carnivorous fish have a relatively short and straight gut and a well-developed stomach with strong gastric acid and digestive enzyme activity [7,8]. In contrast, herbivorous fish have longer and more complex digestive tracts [9]. The digestive tract structure of omnivorous fish is intermediate, situated between those of herbivorous and carnivorous fish [10]. To a certain extent, digestive tract characteristics reflect the physiological traits of ingestion, digestion, and absorption in fish [11]. The gut microbiota is a complex symbiotic community inhabiting the digestive tract, which has co-evolved with the host to form a stable symbiotic relationship [12,13]. Studies have shown that fish guts are colonized by high densities of microorganisms (10^7^–10^11^ CFU/g) [14,15]. The community structure and functional potential of these symbiotic microbes are closely linked to dietary differentiation and play vital roles in host physiological activities, including nutrient metabolism and immune defense, serving as micro-ecological indicators of the host’s feeding habits and physiological state [16,17]. The gut microbiota is co-regulated by endogenous host factors and exogenous environmental factors [18,19]. In particular, environmental shifts triggered by seasonal turnover, fluctuations in food abundance, and changes in host physiological states are key factors driving the seasonal succession of the gut microbiota, significantly altering its diversity and composition [20,21,22,23].

*Hypomesus nipponensis* belongs to the order Salmoniformes, family Osmeridae, genus *Hypomesus,* and is a small cold-water fish native to the freshwater regions of the North Pacific Rim. This species is characterized by biological traits such as an incomplete lateral line, a short life cycle, and early sexual maturity [24,25]. Due to its high economic value and value as a food source, it has been introduced into many water bodies [26]. As an important member of the Osmeridae family, the digestive tract structure of *H. nipponensis* has been preliminarily described, but this research remains limited to general morphological observations of the entire digestive tract [27]. Since its introduction into Bosten Lake in Xinjiang, the population has successfully colonized and adapted, becoming a major target for local fisheries. The histological and ultrastructural characteristics of its esophagus, stomach, and pyloric caeca remain unclear, and how these structures support the fish’s carnivorous diet has not been explored. Regarding its gut microbiota, previous studies characterized the bacterial communities in the gut and skin of *H. nipponensis* from the Neungcheon River in South Korea, revealing that skin microbial diversity (375 OTUs) is significantly higher than that of the gut (250 OTUs), indicating that *H. nipponensis* harbors unique microbial communities in its gut and skin. However, reports on the seasonal succession of its gut microbiota remain lacking [28]. The composition, diversity, and seasonal variation in the gut microbiota of this species in the Bosten Lake environment have not been studied. Therefore, this study combines histological and ultrastructural observations to analyze the microstructural characteristics of various digestive tract segments of *H. nipponensis* and employs 16S rDNA high-throughput sequencing to characterize the composition and dynamics of the gut microbiota in spring, summer, and autumn, with the aim of clarifying the structural basis for its feeding adaptation and the seasonal variation in its gut microbiota, and preliminarily exploring the significance of the relationship between structure and microbial dynamics in host environmental adaptation. The findings will provide a reference for the adaptation of *H. nipponensis* and offer a scientific basis for the management of fishery resources in Bosten Lake.

## 2. Materials and Methods

### 2.1. Sample Collection

Specimens of *H. nipponensis* were collected from Bosten Lake, Bayingolin Mongol Autonomous Prefecture, Xinjiang, China (located between 86°19′50″ E–87°24′50″ E and 41°45′08″ N–42°15′10″ N). All samples were obtained from commercial fishing vessels to minimize disturbance to the habitat. Live fish were immediately transported to the shore in portable tanks with continuous aeration. Prior to processing, all experimental fish were deeply anesthetized with MS-222 (tricaine methanesulfonate) until loss of consciousness [29]. Specimens used for digestive tract structure observation were collected in summer (August 2025), and five individuals were randomly selected for histological observation; various segments of the digestive tract (including esophagus, stomach cardiac, stomach fundic, stomach caeca, pyloric caeca, foregut, midgut, and hindgut) were dissected and fixed in 4% paraformaldehyde solution. Another four individuals were used for scanning electron microscopy observation; corresponding tissue blocks from each segment were fixed in 2.5% glutaraldehyde solution. For gut microbiota analysis, eight individuals were randomly selected from each season. Samples were collected in the following chronological order: summer (August 2024), autumn (November 2024), and spring (May 2025). The entire gut (including its contents) was aseptically removed, immediately frozen in liquid nitrogen, and subsequently transferred to a −80 °C ultra-low temperature freezer (DW-86L388J, Haier, Qingdao, China) for gut microbiota DNA extraction and high-throughput sequencing.

### 2.2. Histological and Ultrastructural Observation of the Digestive Tract

#### 2.2.1. Histological Observation

The samples were dehydrated through a graded ethanol series, cleared in xylene, and embedded in paraffin wax using standard procedures. Serial transverse sections (5–8 μm thick) were cut using a rotary microtome (YD-315, Yidi Medical, Jinhua, China). Sections were stained with hematoxylin and eosin (H & E), mounted with neutral balsam, and observed under a digital microscope (Panthera C2, Motic China Group Co., Ltd., Xiamen, China) equipped with Motic Images Plus imaging software (v3.1, x64) for image acquisition.

#### 2.2.2. SEM Ultrastructural Observation

Tissue blocks (approximately 1 mm × 3 mm) from each segment of the digestive tract were finely trimmed in 2.5% glutaraldehyde solution. After rinsing with 0.1 mol/L phosphate buffer (PBS, pH 7.4), the samples were further fixed in 2.5% glutaraldehyde at 4 °C for 24 h. Subsequently, the samples were rinsed three times with PBS (10 min each), post-fixed with 1% osmium tetroxide for 2 h, and rinsed again with PBS. Dehydration was carried out through a graded ethanol series (30%, 50%, 70%, 80%, 90%, 95%, and 100%; 15 min each), followed by exchange with isoamyl acetate for 20 min and critical point drying. The dried samples were mounted on specimen holders and sputter-coated with gold. Ultrastructural features of the mucosal surfaces along the digestive tract were observed under a scanning electron microscope (Supra 55 VP, Zeiss, Oberkochen Germany) at an accelerating voltage of 2 kV, and representative images were captured.

#### 2.2.3. Morphometric Measurement and Statistical Analysis

Quantitative analysis of tissue sections from each anatomical site was performed using the Motic Images Plus imaging software (v3.1, x64). Observations were made using 4×, 10×, and 40× objective lenses; and the eyepiece magnification was 10×. For each anatomical segment, at least three individuals were selected, with three sections taken from each individual, and 3–15 fields of view were measured per section. The following morphological parameters were directly measured using the software while observing (measurements were performed without blinding): the number of mucosal folds (MN)—the number of mucosal folds per cross-section; mucosal thickness (MT)—the vertical distance from the tip of the mucosal fold to the bottom of the fold invagination; submucosal thickness (SMT)—the vertical distance between the inner and outer boundaries of the submucosa; circular muscle layer thickness (CMT)—the vertical distance between the inner and outer boundaries of the circular muscle layer; and longitudinal muscle layer thickness (LMT)—the vertical distance between the inner and outer boundaries of the longitudinal muscle layer [30]. All measurement data were expressed as the mean ± standard deviation (Mean ± SD). IBM SPSS 27 software (Armonk, NY, USA) was used to perform statistical analyses. For each parameter of each segment, normality was checked using the Shapiro–Wilk test, with *p* < 0.05 indicating deviation from normality, and homoscedasticity was checked using Levene’s test, with *p* < 0.05 indicating violation of homoscedasticity. Depending on the results, one-way ANOVA followed by Tukey’s HSD was used for normal and homoscedastic data; whereas one-way ANOVA followed by the Games–Howell test was used for normal but heteroscedastic data. *p* < 0.05 was considered statistically significant.

### 2.3. Gut Microbiota Analysis Based on 16S rDNA Gene Sequencing

#### 2.3.1. Microbial DNA Extraction

Total microbial DNA was extracted from gut content samples of *H. nipponensis* using the MagPure Stool DNA KF Kit B (MAGEN, Guangzhou, China) according to the manufacturer’s instructions. The extracted DNA was stored at −80 °C for subsequent analysis.

#### 2.3.2. 16S rDNA Gene Amplification and Sequencing

Libraries were prepared using 2× Phanta Max Master Mix (VAZYME, Nanjing, China). The hypervariable V3–V4 region of the bacterial 16S rDNA gene was amplified using universal primers 338F (5′-ACTCCTACGGGAGGCAGCAG-3′) and 806R (5′-GGACTACHVGGGTWTCTAAT-3′) with attached sequencing adapters. PCR enrichment was performed in a 50 μL reaction containing 30 ng template and fusion PCR primers. PCR cycling conditions were as follows: 95 °C for 3 min; 30 cycles of 95 °C for 15 s, 56 °C for 15 s, 72 °C for 45 s; and a final extension at 72 °C for 5 min. PCR products were purified using DNA magnetic beads (LB00V60 BGI, Shenzhen, China). Next, the final double-stranded library products were denatured to generate single-stranded library products. Then, the circularization reaction was set up to obtain single-stranded circularized DNA products. Any single-stranded linear DNA was digested and removed. The final single-stranded circularized library was amplified with phi29 and rolling circle amplification (RCA) to generate DNA nanoballs (DNBs) carrying multiple copies of the initial single-stranded library molecule. The DNBs were loaded into the patterned nanoarray, and sequencing reads of PE300 base length were generated using the DNBSEQ-G400 platform (DNBSEQ-G400, BGI, Shenzhen, China).

#### 2.3.3. Sequence Processing and Taxonomic Assignment

Primer and adapter sequences were removed from raw reads using Cutadapt (v2.6). Subsequent stringent quality filtering was performed with iTools Fqtools: sequences were trimmed using a sliding window (window size: 30 bp; average quality threshold: Q20). Reads shorter than 75% of the original length, containing ambiguous bases (N), or exhibiting low complexity (e.g., homopolymers ≥ 10 bp) were removed to obtain clean data [31]. Denoising was performed using DADA2 in QIIME2 to obtain amplicon sequence variants (ASVs) at 100% similarity. Representative ASV sequences were taxonomically annotated using the RDP Classifier (v2.2) against the RDP 16S rDNA database with a confidence threshold of 0.6. ASVs not reliably assigned to the bacterial domain were removed. The resulting high-quality bacterial ASVs were used for all downstream analyses. All samples were rarefied to the minimum sequence count across all samples to normalize sequencing depth. A total of 24 gut samples of *H. nipponensis* were collected from Bosten Lake (8 samples per season for spring, summer, and autumn). The raw sequencing data have been deposited in the NCBI database. The accession number is PRJNA1435073.

#### 2.3.4. Microbial Community Diversity Analysis

Alpha diversity indices, including the Sob, Chao, Ace, Shannon, Simpson and Coverage indices, were calculated for each sample using mothur (v1.31.2) [32]. Rarefaction curves were generated to assess sequencing depth. For each alpha diversity index, we first tested normality (Shapiro–Wilk) and homoscedasticity (Levene). Depending on the results, we used one-way ANOVA with Tukey HSD for normal and homoscedastic data, one-way ANOVA with the Games–Howell test for normal but heteroscedastic data, or the Kruskal–Wallis test with Dunn’s Bonferroni correction for non-normal data. A threshold of *p* < 0.05 was considered statistically significant. Inter-group comparisons and visualization were performed using GraphPad Prism (v10).

#### 2.3.5. Analysis of Gut Microbiota Composition Differences

To assess differences in gut microbiota composition among seasons, the relative abundances of major bacterial phyla and genera were compared. Statistical analysis was performed using IBM SPSS Statistics 27.0. The Kruskal–Wallis test was employed, and a significance level of *p* < 0.05 was considered statistically significant.

#### 2.3.6. Differential Abundance Analysis and Functional Prediction

Biomarker identification: The Linear Discriminant Analysis Effect Size (LEfSe) method was applied to identify taxa with significantly different abundances between groups using the BGI online platform (https://meta.bgi.com) (accessed on 15 December 2025), with thresholds set at an LDA score > 4.0 and *p* < 0.05. Functional prediction: The functional potential of the gut microbial communities was inferred from 16S rDNA gene sequences using PICRUSt2 (v2.3.0-b) and R (v3.4.10). Predicted gene families were mapped to the Kyoto Encyclopedia of Genes and Genomes (KEGG) pathway database. The Kruskal–Wallis test was employed, and a significance level of *p* < 0.05 was considered statistically significant.

## 3. Results

### 3.1. Histological Structure of the Digestive Tract of Hypomesus nipponensis

**Figure 1:**
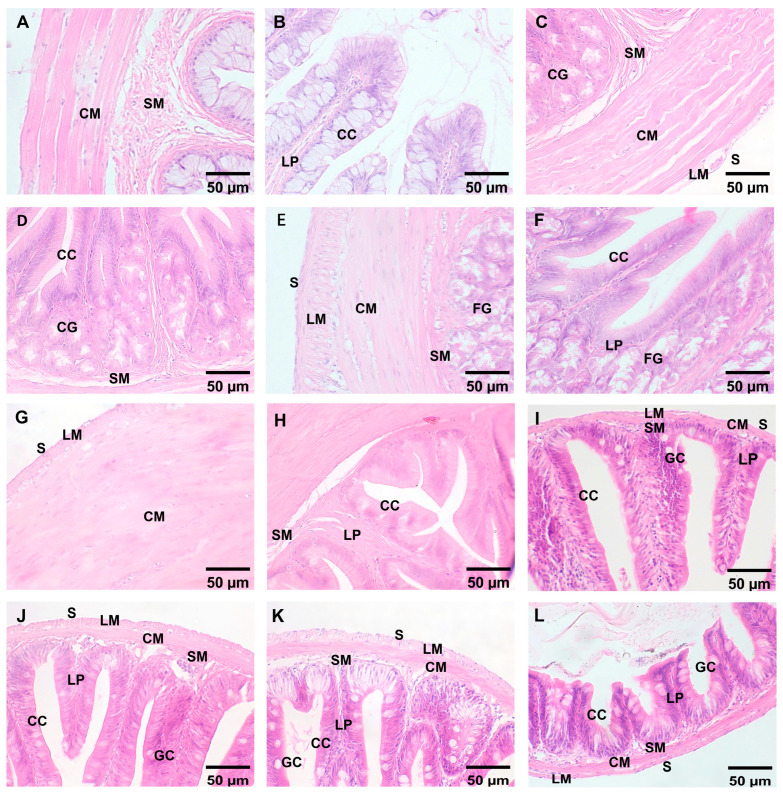
H&E staining of the digestive tract tissue of *H. nipponensis* (40×). Note: (**A**,**B**) esophagus; (**C**,**D**) stomach cardiac; (**E**,**F**) stomach fundic; (**G**,**H**) stomach caeca; (**I**) pyloric caeca; (**J**) foregut; (**K**) midgut; (**L**) hindgut; Scale bar, 50 μm; S: serosa; CM: circular muscle layer; LM: longitudinal muscle layer; SM: submucosa; LP: lamina propria of the mucosa; CC: columnar cells; GC: goblet cells; CG: cardiac glands; FG: gastric glands.

The results showed that these eight parts were composed of the mucosal layer, submucosal layer, muscular layer, and serosal layer from the inside out. The esophageal mucosal layer of *H. nipponensis* was composed of stratified columnar epithelial cells, and goblet cells could be seen between the columnar epithelial cells. The epithelial cells at the top of the mucosal layer were arranged more closely than those at the bottom (Figure 1A,B). The number of mucosal folds in the esophagus was 22.89 ± 3.37, which was significantly more than that in the cardiac part (*p* < 0.05), but the height of the mucosal folds was 181.46 ± 29.54 μm, which was significantly lower than that in the cardiac and body parts (*p* < 0.05). The submucosal layer was loose connective tissue with a large number of blood vessels, and its thickness was 35.43 ± 16.29 μm. The muscular layer was composed of striated muscle, and the circular muscle layer was well developed, with a thickness of 85.67 ± 21.94 μm, but the longitudinal muscle layer was not well developed (Table 1).

The histological staining results of the stomach of *H. nipponensis* showed that it could also be divided into four layers from the inside out. The innermost mucosal layer was indented inward to form gastric pits, which were composed of single-layer columnar epithelial cells (Figure 1E,F). The epithelial cells were closely arranged, with the nuclei neatly located at the bottom of the cells and deeply stained. Mucous cells were distributed, and the lamina propria contained a large number of gastric glands. In the middle of the stomach body, the mucosal folds showed a phenomenon of branch fusion, and the mucosal layer appeared in sheets, greatly increasing the area of the lamina propria, and the number of gland cells further increased. The comparison of histological feature measurement results showed that the height of the mucosal folds in the stomach body was significantly higher than that in the pyloric part (219.79 ± 60.21 μm) (*p* < 0.05) (Table 1). The thickness of the circular muscle layer in the stomach body (135.88 ± 32.59 μm), cardiac part (108.74 ± 23.38 μm), and pyloric part (244.84 ± 49.01 μm) was significantly higher than that of the longitudinal muscle layer (*p* < 0.05). In addition, the thickness of the circular muscle layer in the pyloric part was 244.84 ± 49.01 μm, which was the thickest part of the circular muscle layer in the entire digestive tract, significantly higher than that in the esophagus, cardiac part, and stomach body (*p* < 0.05).

The number of pyloric caeca folds was relatively low at 11.33 ± 2.52, significantly lower than the number of mucosal folds in the gut region (*p* < 0.05). The height of the developed mucosal layer was 179.28 ± 33.11 μm (Table 2). Its structure was similar to that of the gut, with folds composed of a single layer of columnar epithelial cells, containing goblet cells. The submucosa and muscularis were not well-developed (Figure 1I). The thickness of the submucosa was 3.88 ± 1.48 μm, and the thickness of the circular muscle layer of the muscularis was 5.84 ± 1.09 μm, which was significantly lower than that of the midgut (10.46 ± 1.37 μm, *p* < 0.05), while the thickness of the longitudinal muscle layer was 3.67 ± 1.53 μm, with no significant difference (*p* > 0.05).

The gut mucosa was well-developed and formed abundant folds inward, composed of a single layer of columnar epithelial cells, with numerous round goblet cells between the epithelial cells (Figure 1J–L). The number of folds in the foregut was the highest at 28.42 ± 3.63, and the height was 188.69 ± 20.03 μm. The midgut was similar to the foregut, where the height of the mucosal layer in the midgut was 144.72 ± 40.49 μm, and the number of folds was 27.50 ± 1.23. The height of the mucosal layer in the hindgut was 106.14 ± 14.88 μm, and the number of folds was 22.22 ± 0.84. The difference between the circular muscle and longitudinal muscle thicknesses in the foregut reached statistical significance (*p* < 0.05) (Table 2). Compared with the foregut, the number and height of the mucosal layer in the hindgut were significantly reduced (*p* < 0.05). Submucosal thickness and longitudinal muscle thickness showed no significant differences among gut segments (*p* > 0.05).

### 3.2. Scanning Electron Microscopy of the Digestive Tract of H. nipponensis

**Figure 2:**
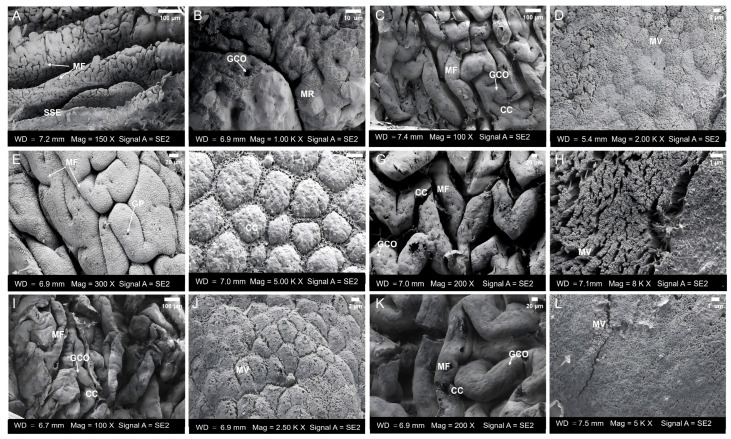
Scanning electron microscope observation of the digestive tract of *H. nipponensis.* Note: (**A**,**B**) Esophagus. (**C**,**D**) Foregut. (**E**,**F**) Stomach. (**G**,**H**) Midgut. (**I**,**J**) Pyloric caeca. (**K**,**L**) Hindgut. MF: Mucosal folds; SSE:Stratified squamous epithelium; MR: Microridges; GCO: Goblet cell openings; CC: Columnar epithelial cells; MV: Microvilli; GP: Gastric pits.

Under a scanning electron microscope, the esophageal mucosa of *H. nipponensis* exhibited well-developed longitudinal folds arranged in parallel, with secondary mucosal folds. The epithelial surface was uneven and covered with tightly arranged flattened epithelial cells resembling scales. Secretory pores were depressed into pits between the epithelial cells and were clearly visible (Figure 2A). Dense wave-like microridges arranged in a fingerprint-like pattern were observed on the surface of the epithelial cells (Figure 2B). The gastric mucosa of *H. nipponensis* displayed well-developed convoluted and twisted folds. Gastric pits were densely distributed on the mucosal surface, where the epithelial cells were polygonal and tightly arranged, and the cell surfaces were covered with abundant mucus (Figure 2E,F).

The mucosal folds of the pyloric caeca were well-developed and formed a complex interlacing network. Goblet cell openings were scattered on the mucosal surface, and the epithelium consisted of simple columnar cells with microvilli covering the apical surface (Figure 2I,J). The mucosal surfaces of the foregut, midgut, and hindgut of *H. nipponensis* all exhibited well-developed mucosal folds with irregular shapes. Goblet cell openings were scattered, and the mucosal epithelium was composed of simple columnar cells with abundant microvilli covering the apical surface (Figure 2C,D,G,H,K,L). The density of microvilli in the gut epithelium ranged from approximately 67.96 to 87.25 per μm^2^, while that in the pyloric caeca ranged from approximately 52.32 to 139.91 per μm^2^. Distinct differences in mucosal surface structure were observed along the digestive tract of *H. nipponensis*, collectively forming an efficient digestive and absorptive system.

### 3.3. Composition and Diversity of the Gut Microbiota of H. nipponensis

#### 3.3.1. Analysis of Gut Microbiota ASVs in *H. nipponensis*

**Figure 3:**
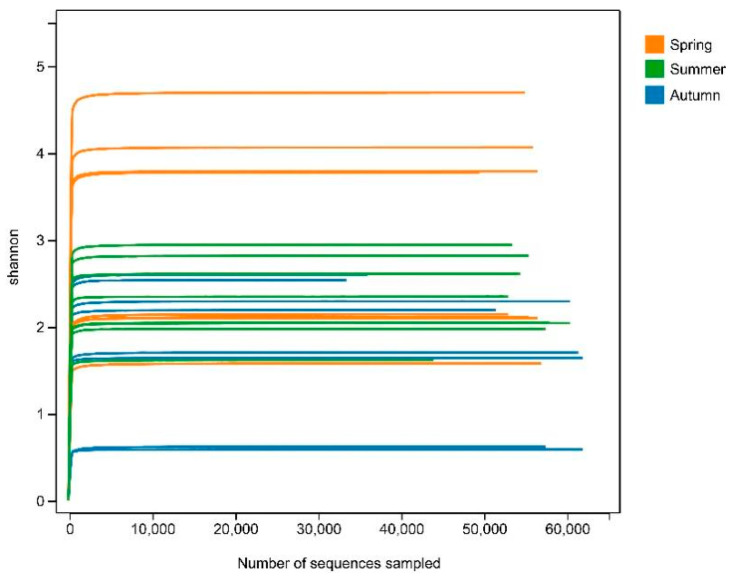
Rarefaction curves of the Shannon index of gut microbiota of *H. nipponensis* across different seasons.

A total of 1,568,939 raw reads were obtained from 24 gut samples of *H. nipponensis* from Bosten Lake, with an average of 65,372 reads per sample. After quality filtering and denoising using DADA2, 1,305,876 high-quality clean reads were retained, averaging 54,411 reads per sample, with an average effective ratio of 84%. The sequence quality met the requirements for subsequent bioinformatics analyses (Table 3). Rarefaction curve analysis showed that the Shannon index plateaued with increasing sequencing depth, indicating that the sequencing depth adequately covered the majority of microbial diversity in the samples (Figure 3).

**Figure 4:**
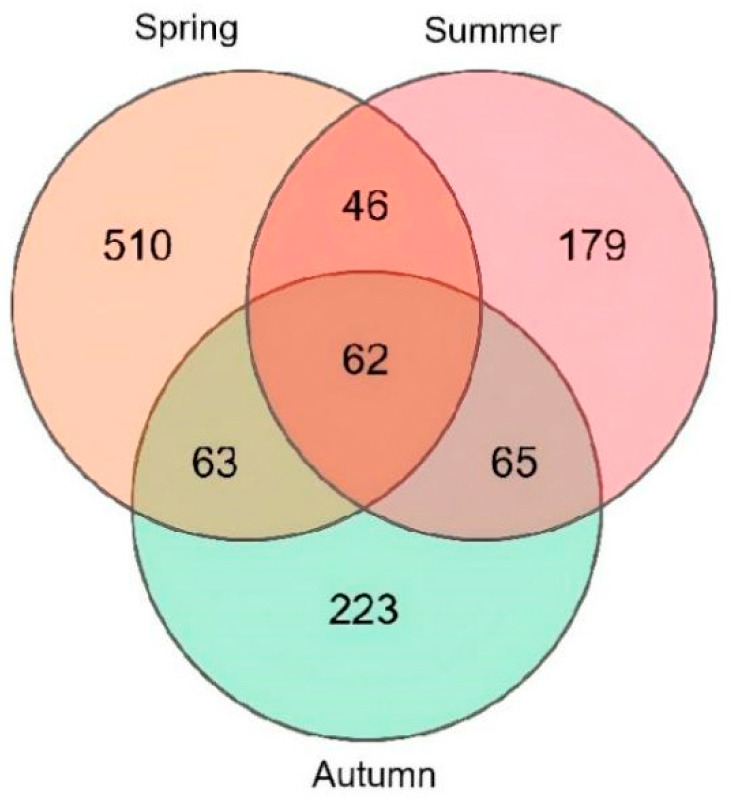
Venn diagram showing the distribution of gut microbiota ASVs in *H. nipponensis* across different seasons.

Based on 100% sequence similarity, a total of 1148 featured amplicon sequence variants (ASVs) were obtained from all samples. Venn diagram results revealed differences in the ASV composition of the gut microbiota across seasons: spring exhibited 510 unique ASVs, summer had 179 unique ASVs, and autumn possessed 223 unique ASVs. The number of ASVs shared among all three seasons was 62. Pairs of seasons shared the following ASV numbers: 46 between spring and summer, 63 between spring and autumn, and 65 between summer and autumn. The number of unique members within the microbiota varied considerably across different seasons, while the proportion of shared members was relatively low (Figure 4).

#### 3.3.2. Analysis of Gut Microbiota Alpha Diversity in *H. nipponensis* Across Different Seasons

**Figure 5:**
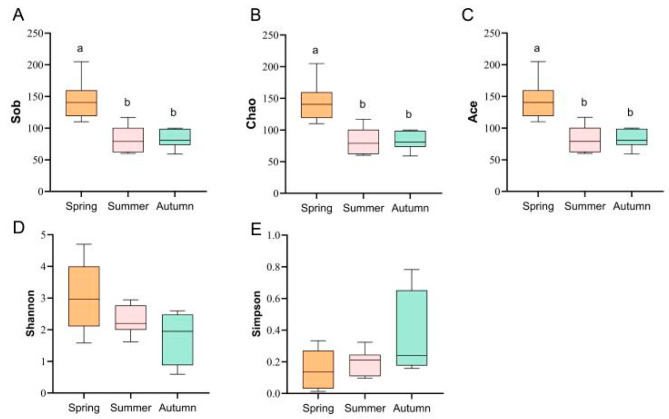
Alpha diversity indices of the gut microbiota in *H. nipponensis* across different seasons. Note: (**A**) Sob; (**B**) Chao; (**C**) Ace; (**D**) Shannon; (**E**) Simpson. Different lowercase letters above boxes indicate significant differences among groups (*p* < 0.05), while the same letters indicate no significant difference (*p* > 0.05).

To assess the impact of seasonal variation on the gut microbial community diversity of *H. nipponensis*, alpha diversity indices including Sob, Chao, and Ace, as well as Shannon and Simpson, were calculated for samples from spring, summer, and autumn (Figure 5). The results showed that the Sob, Chao, and Ace indices in spring were significantly higher than those in summer and autumn (*p* < 0.05), while no significant differences were observed between summer and autumn (*p* > 0.05). The Shannon index was slightly higher in spring compared to summer and autumn, but the differences among the three seasons were not statistically significant (*p* > 0.05). The Simpson index showed no significant differences among groups (*p* > 0.05), with autumn exhibiting slightly higher values than spring and summer. Seasonal differences in alpha diversity were primarily driven by changes in species richness, which peaked in spring, whereas evenness and dominance remained relatively stable across seasons.

#### 3.3.3. Composition Analysis of the Gut Microbiota at the Phylum and Genus Levels

**Figure 6:**
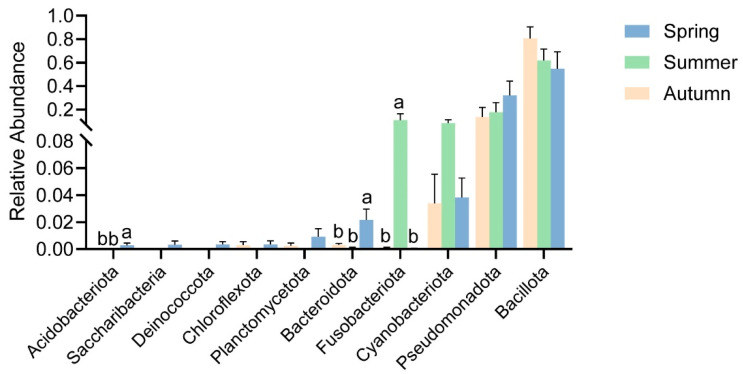
Gut microbiota composition of *H. nipponensis* at the phylum level across different seasons. Note: Bars represent the mean relative abundance (±SE). Different lowercase letters above bars indicate significant differences among seasons within each phylum (*p* < 0.05).

The relative abundances of the absolute dominant phyla, Bacillota and Pseudomonadota, remained stable across seasons, and the relative abundances of other phyla in the gut microbiota of *H. nipponensis* exhibited certain seasonal variations (Figure 6). Specifically, the relative abundances of Fusobacteriota in summer were significantly higher than those in spring and autumn (*p* < 0.05), with no significant difference between spring and autumn (*p* > 0.05). The relative abundance of Actinomycetota in spring was significantly higher than that in summer and autumn (*p* < 0.05), while no significant difference was observed between summer and autumn (*p* < 0.05). For Bacteroidota, its relative abundance in spring was significantly higher than that in summer (*p* < 0.05).

**Figure 7:**
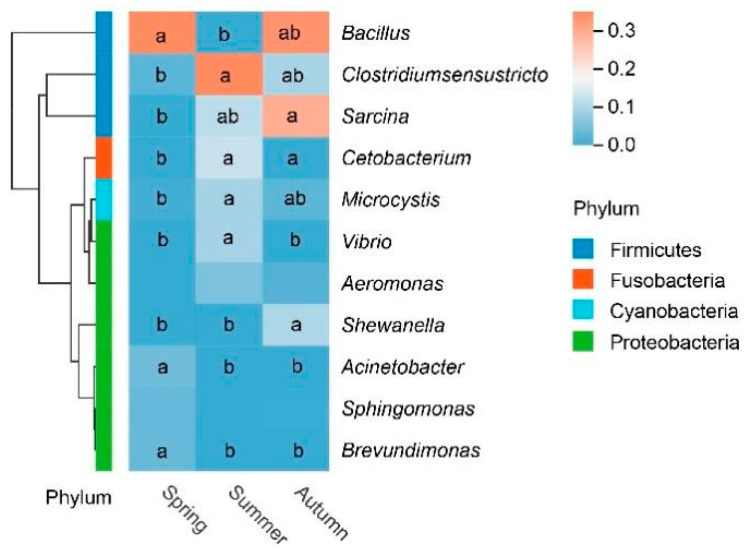
Gut microbiota composition of *H. nipponensis* at the genus level across different seasons. Different lowercase letters in the same row indicate significant differences (*p* < 0.05).

Based on a relative abundance threshold of 0.01, a total of 11 dominant bacterial genera were identified. The differential analysis results showed that *Acinetobacter* and *Brevundimonas* genera in the gut microbiota of *H. nipponensis* had significantly higher abundances in spring compared to summer and autumn (*p* < 0.05), while there were no significant differences between summer and autumn (*p* > 0.05) (Figure 7). *Bacillus* had higher abundances in spring and autumn, with the abundance in spring being significantly higher than that in summer (*p* < 0.05), but no significant difference between summer and autumn were observed (*p* > 0.05). In summer, *Vibrio* had significantly higher abundances than spring and autumn (*p* < 0.05), while the abundances of *Clostridium sensu stricto*, *Microcystis*, and *Cetobacterium* in summer were significantly higher than those in spring (*p* < 0.05), and there was no significant difference between summer and autumn (*p* > 0.05). The abundance of *Shewanella* in autumn was significantly higher than that in spring and summer (*p* < 0.05); the abundance of *Sarcina* was significantly higher than that in spring (*p* < 0.05), but there was no significant difference between summer and autumn (*p* > 0.05). There were no significant differences in the abundances of *Sphingomonas* and *Aeromonas* among spring, summer, and autumn (*p* > 0.05).

#### 3.3.4. LEfSe Analysis of Differential Gut Microbiota in *H. nipponensis* Across Different Seasons

**Figure 8:**
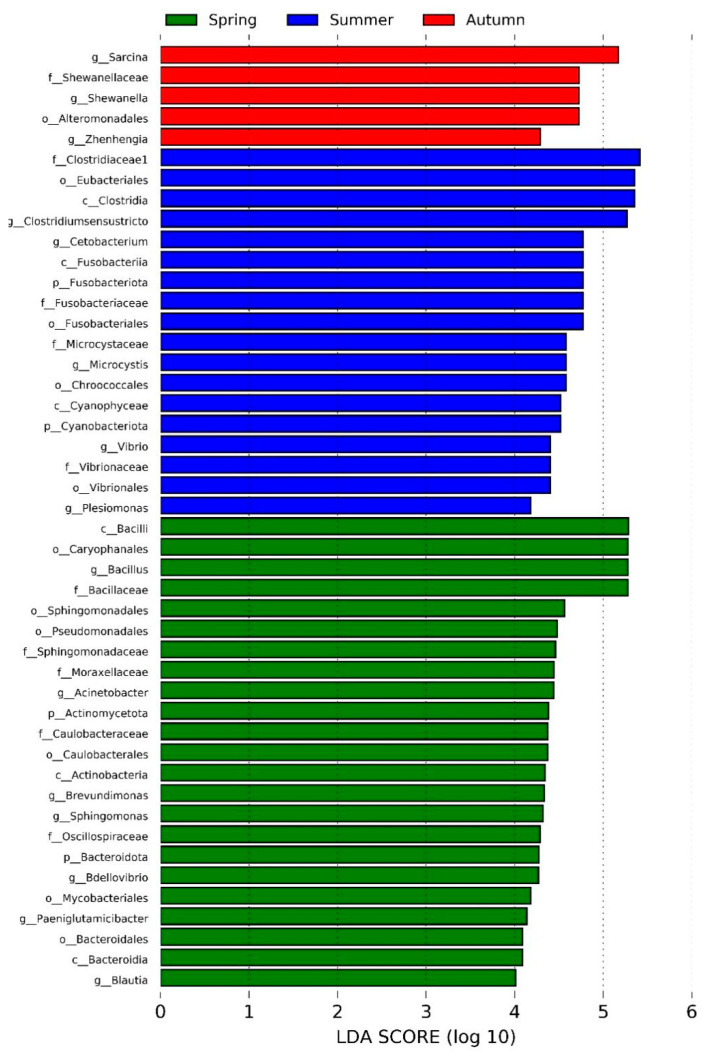
Linear discriminant analysis (LDA) of gut microbiota composition of *H. nipponensis* across different seasons. (LDA score > 4, *p* < 0.05).

LEfSe analysis with an LDA score threshold of 4.0 (*p* < 0.05) revealed distinct sets of taxonomic biomarkers for each season. A total of 23, 18, and 5 differential taxa were identified in the spring, summer, and autumn groups, respectively (Figure 8). In spring, the enriched taxa spanned multiple taxonomic levels. These included the phyla Bacillota, Actinomycetota, and Bacteroidota, along with the classes Bacilli and Actinobacteria. At the order level, Caryophanales, Caulobacterales, Sphingomonadales, and Pseudomonadales were overrepresented. Corresponding families included Bacillaceae, Caulobacteraceae, and Sphingomonadaceae, while the key genera were *Bacillus*, *Acinetobacter*, *Sphingomonas*, and *Brevundimonas*. For summer, the signature taxa comprised the phyla Fusobacteriota, Cyanobacteriota, and Bacillota. The classes Fusobacteriia, Cyanophyceae, and Clostridia were also enriched, as were the orders Fusobacteriales, Chroococcales, Eubacteriales, and Vibrionales. Several families contributed to this signal, including Fusobacteriaceae, Microcystaceae, Clostridiaceae 1, and Vibrionaceae, with the genera *Cetobacterium*, *Microcystis*, *Clostridium sensu stricto*, and *Vibrio* showing elevated abundances. In autumn, the number of differentially abundant taxa was considerably lower. The order Alteromonadales emerged as a biomarker, along with the families Shewanellaceae. At the genus level, *Shewanella*, *Sarcina*, and *Zhenhengia* were the main discriminators.

#### 3.3.5. Functional Differences in Gut Microbiota of *H. nipponensis* Across Seasons

**Figure 9:**
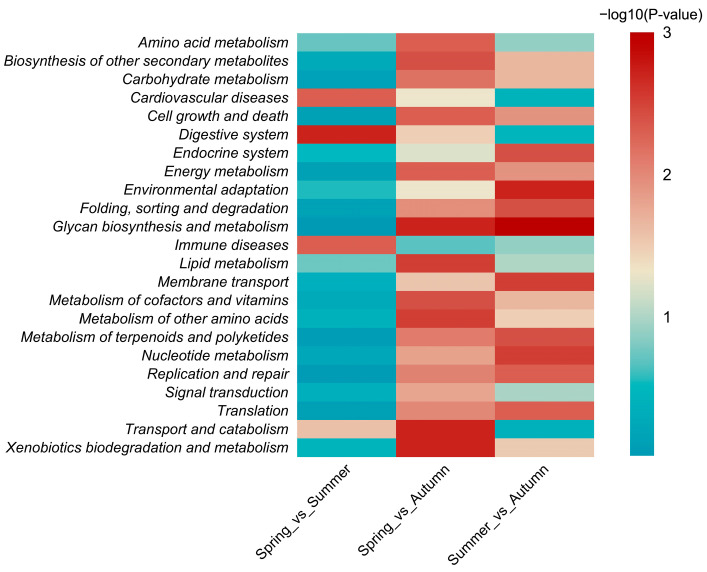
Differential metabolic pathways predicted by KEGG in the gut microbiota of *H. nipponensis* across different seasons.

Based on PICRUSt2 functional prediction from 16S rDNA gene sequences, a total of 33 KEGG pathways at level 2 were annotated. The functional profiles of the gut microbiota of *H. nipponensis* showed marked differences across seasons, with 23 pathways differing significantly among spring, summer, and autumn (*p* < 0.05) (Figure 9). The differences between spring and autumn were the most prominent, with spring showing significantly higher abundances in pathways related to amino acid metabolism, carbohydrate metabolism, energy metabolism, cell growth and death, translation, and transport and catabolism than autumn (*p* < 0.05). Fewer differences were observed between spring and summer; nevertheless, spring had significantly higher abundances in pathways such as the digestive system than summer (*p* < 0.05). In contrast, summer exhibited significantly higher abundances in pathways including glycan biosynthesis and metabolism, membrane transport, nucleotide metabolism, replication and repair, and translation than autumn (*p* < 0.05). Overall, spring displayed the most active functional profile in the gut microbiota.

## 4. Discussion

The structural differentiation of the esophagus and stomach in *H. nipponensis* reflects its high degree of adaptation to a carnivorous feeding mode. The esophagus was short and thick, with the muscular layer composed of striated muscle and a well-developed circular muscle layer (85.67 ± 21.94 μm), which provided a structural basis for esophageal expansion [10]. The mucosal layer consisted of stratified columnar epithelium rich in goblet cells, which secreted mucins to form a lubricating layer, thereby reducing frictional damage from hard food [33]. The stomach was V-shaped, with well-developed gastric glands in the cardiac and fundic regions. The mucosal fold heights in these regions reached 329.34 ± 73.32 μm and 362.88 ± 39.90 μm, respectively, and the folds exhibited branching and fusion to form sheet-like structures, thereby enhancing the secretory capacity for pepsinogen and hydrochloric acid [34]. The circular muscle layer in the pyloric region was significantly thickened (244.84 ± 49.01 μm), forming the pyloric sphincter to precisely regulate the gastric emptying rate [35]. These structures collectively constituted an efficient digestive front end, providing a morphological basis for the prehension and digestion of hard food by *H. nipponensis*.

The pyloric caeca and the gut together formed a digestive and absorptive structure that supported the nutritional demands of *H. nipponensis*. Previous studies have shown that the pyloric caeca play a key role in lipid absorption [36]. In the present study, the pyloric caeca possessed a well-developed mucosal layer (179.28 ± 33.11 μm), and the epithelial cell surfaces were covered with high-density microvilli (52.32–139.91/μm^2^), which are typical structural features of regions involved in enzymatic digestion and nutrient absorption. In addition, the mucosal surfaces of all gut segments in *H. nipponensis* were covered with dense microvilli, expanding the absorptive surface area and playing important roles in lipid and protein digestion [7,9].

This study systematically analyzed the composition and functional characteristics of the gut microbiota of *H. nipponensis* in Bosten Lake using 16S rDNA high-throughput sequencing. At the phylum level, Bacillota and Pseudomonadota were the absolute dominant groups, further supporting the classic view that these two phyla are the primary colonizers of the fish gut [14,15]. This composition was highly consistent with studies on *H. nipponensis* in South Korea, which also identified Pseudomonadota (51.5%) and Bacillota (30.6%) as dominant, followed by Bacteroidota (7.7%) and Actinomycetota (5.2%) [28,29], reflecting the stability of the core microbial composition in this species. Further comparison revealed that species richness indices were significantly higher in spring than in summer and autumn (*p* < 0.05).

LEfSe analysis identified 23, 18, and 5 differential taxonomic units for spring, summer, and autumn, respectively. These seasonal variations were closely associated with dietary fluctuations. As a carnivorous fish, *H. nipponensis* primarily feeds on zooplankton [37]. Research has shown that in Lake Ulungur, the diet of *H. nipponensis* was more diverse in spring, with rotifers accounting for 69.49% of the numerical percentage (N%), alongside surface insects (11.46%), chironomid larvae (8.97%), copepods (6.44%), and cladocerans (1.69%). In contrast, cladocerans accounted for 96.58% and 98.89% of the diet in summer and autumn, respectively, representing extreme dietary specialization [37]. While the diverse feeding patterns observed in spring increased microbial richness by providing niches for rare taxa, these findings differed notably from those of sticklebacks and perch, which suggested that generalist feeders possess lower microbial diversity than specialists [38].

At the genus level, the composition and function of the microbiota showed significant seasonal differentiation. Spring was characterized by *Bacillus*, which possesses broad metabolic capabilities. Summer shifted toward protein-fermenting bacteria such as *Clostridium* and *Cetobacterium*, which are involved in amino acid metabolism and short-chain fatty acid production [39]. Autumn was marked by *Shewanella* and *Sarcina*, with *Shewanella* being associated with chitin degradation [40,41]. Although the zooplankton consumed in summer were protein-rich, components such as glycogen may induce the microbiota to maintain high levels of carbohydrate utilization to ensure basal energy metabolism [42,43]. Although the microstructural observation of the digestive tract was limited to a single season, it still provided a histological reference for understanding the adaptation between the digestive tract structure and feeding habits of *H. nipponensis*, and the stable intestinal environment provided a basis for microbial colonization. This study preliminarily revealed the seasonal succession pattern of the gut microbiota in *H. nipponensis* from Bosten Lake and its adaptation to feeding habits. However, the direct causal relationship between the structure and the seasonal dynamics of the gut microbiota remains to be further experimentally validated.

## 5. Conclusions

*H. nipponensis* possesses a digestive tract morphologically adapted to carnivory, providing a structural basis for nutrient absorption and microbial colonization. The gut microbiota exhibited clear seasonal variation: species richness indices (Chao, Ace, Sob) were significantly higher in spring than in summer and autumn; the dominant marker genera in spring, summer, and autumn were *Bacillus*, *Clostridium*, and *Sarcina*, respectively. However, due to the seasonal limitation of the histological data, the relationship between gut structural features and seasonal changes in the gut microbiota remains to be further elucidated. Furthermore, future studies integrating synchronized water physicochemical parameters are needed to more comprehensively identify the core environmental drivers underlying the seasonal fluctuations in the gut microbiota in *H. nipponensis*. Nevertheless, this study provides baseline histological and microbiological data for the ecological adaptation of this fish in Bosten Lake and a scientific reference for the management of local fishery resources.

## Figures and Tables

**Table 1:** Histological features of the esophagus and stomach in *H. nipponensis*.

Parameter (μm)	Esophagus	Stomach Cardiac	Stomach Fundic	Stomach Caeca	*p*
MN (*n*)	22.89 ± 3.37 ^a^	8.56 ± 0.69 ^b^	15.58 ± 3.81 ^ab^	21.13 ± 6.83 ^a^	0.012
MT	181.46 ± 29.54 ^c^	329.34 ± 73.32 ^ab^	362.88 ± 39.90 ^a^	219.79 ± 60.21 ^bc^	0.002
SMT	35.43 ± 16.29 ^ab^	14.48 ± 1.65 ^b^	25.78 ± 3.73 ^a^	18.60 ± 5.80 ^ab^	0.034
CMT	85.67 ± 21.94 ^b^	108.74 ± 23.38 ^Ab^	135.88 ± 32.59 ^Ab^	244.84 ± 49.01 ^Aa^	0.000
LMT	—	17.54 ± 1.72 ^Ba^	27.11 ± 5.44 ^Ba^	19.40 ± 5.75 ^Ba^	0.61
*p* (CMT vs. LMT)		0.022	0.006	0.000	

Note: MN, mucosal number; MT, mucosal thickness; SMT, submucosal thickness; CMT, circular muscle thickness; LMT, longitudinal muscle thickness. Different lowercase letters in the same row indicate significant differences among different segments (*p* < 0.05). Different uppercase letters indicate significant differences between CMT and LMT within the same segment (*p* < 0.05). “—” indicates that the data could not be reliably measured.

**Table 2:** Histological features of the pyloric caeca and gut in *H. nipponensis*.

Parameter (μm)	Pyloric Caeca	Foregut	Midgut	Hindgut	*p*
MN (*n*)	11.33 ± 2.52 ^c^	28.42 ± 3.63 ^a^	27.50 ± 1.23 ^ab^	22.22 ± 0.84 ^b^	0.000
MT	179.28 ± 33.11 ^ab^	188.69 ± 20.03 ^a^	144.72 ± 40.49 ^ab^	106.14 ± 14.88 ^b^	0.019
SMT	3.88 ± 1.48 ^a^	6.46 ± 3.48 ^a^	6.72 ± 1.84 ^a^	5.06 ± 1.79 ^a^	0.422
CMT	5.84 ± 1.09 ^Ab^	12.84 ± 4.60 ^Aab^	10.46 ± 1.37 ^Aa^	6.88 ± 2.27 ^Aab^	0.031
LMT	3.67 ± 1.53 ^Aa^	6.87 ± 2.50 ^Ba^	9.46 ± 3.25 ^Aa^	5.95 ± 2.07 ^Aa^	0.075
*p* (CMT vs. LMT)	0.092	0.041	0.443	0.203	

Note: MN, mucosal number; MT, mucosal thickness; SMT, submucosal thickness; CMT, circular muscle thickness; LMT, longitudinal muscle thickness. Different lowercase letters in the same row indicate significant differences among different segments (*p* < 0.05). Different uppercase letters indicate significant differences between CMT and LMT within the same segment (*p* < 0.05).

**Table 3:** Sequencing results and sequence information of *H. nipponensis* samples.

Group	Sample	Raw Reads	Clean Reads	(Clean Read/ Raw Read) × 100%	Coverage
Spring	f1	67,752	56,979	84	1
	f2	68,125	53,437	78	1
	f3	67,427	55,745	83	1
	f4	67,504	57,278	85	1
	f5	67,923	49,574	73	1
	f6	67,747	56,396	83	1
	f7	67,414	56,924	84	1
	f8	67,246	55,107	82	1
Summer	e1	56,569	44,139	78	1
	e2	67,478	60,821	90	1
	e3	68,104	53,788	79	1
	e4	68,143	57,528	84	1
	e5	68,168	58,072	85	1
	e6	67,302	55,803	83	1
	e7	67,555	53,278	79	1
	e8	67,791	54,761	81	1
Autumn	n1	42,995	33,633	78	1
	n2	47,905	36,244	76	1
	n3	68,041	57,789	85	1
	n4	67,407	61,640	91	1
	n5	67,484	62,298	92	1
	n6	67,667	51,879	77	1
	n7	67,896	62,147	92	1
	n8	67,296	60,616	90	1
Total		1,568,939	1,305,876	84	1
Mean		65,372	54,411	84	1

## Data Availability

The raw sequencing data supporting the conclusions of this article have been deposited in the NCBI Sequence Read Archive (SRA) under BioProject accession number PRJNA1435073. The data are currently under embargo and will be made publicly available upon publication of the manuscript.

## Abbreviations

The following abbreviations are used in this manuscript:

ASVs	Amplicon Sequence Variants
HE	Hematoxylin and Eosin
*H. nipponensis*	*Hypomesus nipponensis*
KEGG	Kyoto Encyclopedia of Genes and Genomes
LEfSe	Linear Discriminant Analysis Effect Size
OTU	Operational Taxonomic Unit
PTS	Phosphotransferase System
SD	Standard Deviation
SE	Standard Error
SEM	Scanning Electron Microscopy
μm	Micrometer

## References

[1] Ni L., Wu X., Li F., Zou Q., Du J., Lai J., Liu Y. (2025). Morphological Analysis of the Intestine in Yangtze Sturgeon (*Acipenser dabryanus*) During Development. Fishes.

[2] Kasozi N., Iwe Degu G., Mukalazi J., Kato C.D., Kisekka M., Owori Wadunde A., Kityo G., Namulawa V.T. (2017). Histomorphological description of the digestive system of pebbly fish, *Alestes baremoze* (Joannis, 1835). Sci. World J..

[3] Teresa O., Maciej K. (2019). Digestive System. The Histology of Fishes.

[4] Liu H., Zhang Y. (2001). The anatomy on the digestive system of *Silurus meridionalis*. J. Quanzhou Norm. Univ. Nat. Sci..

[5] Wilson J.M., Castro L.F.C. (2010). Morphological diversity of the gastrointestinal tract in fishes. Fish Physiology.

[6] Amundsen P.A., Sánchez-Hernández J. (2019). Feeding studies take guts—Critical review and recommendations of methods for stomach contents analysis in fish. J. Fish Biol..

[7] Jiao F., Zhang L., Limbu S.M., Yin H., Xie Y., Yang Z., Shang Z., Kong L., Rong H. (2023). A comparison of digestive strategies for fishes with different feeding habits: Digestive enzyme activities, intestinal morphology, and gut microbiota. Ecol. Evol..

[8] Rincón L., Redondo F., Kobrinsky W., Pandolfi M., Pozzi A.G. (2023). Morphological study of the digestive tract of the cardinal tetra, *Paracheirodon axelrodi* (Characiformes: Characidae). Neotrop. Ichthyol..

[9] Johnson K.S., Clements K.D. (2022). Histology and ultrastructure of the gastrointestinal tract in four temperate marine herbivorous fishes. J. Morphol..

[10] Neves M.P., Amorim J.P.d.A., Delariva R.L., Kratina P., Fialho C.B. (2024). Linking anatomical and histological traits of the digestive tract to resource consumption and assimilation of omnivorous tetra fishes. Ecol. Evol..

[11] Xu G., Chen X., Du J., Mou Z. (2009). Fish Digestive System: It’s Structure, Function and The Distrbutions and Characteristics of Digestive Enzymes. Chin. J. Fish..

[12] Ganguly S., Prasad A. (2012). Microflora in fish digestive tract plays significant role in digestion and metabolism. Rev. Fish Biol. Fish..

[13] McFall-Ngai M., Hadfield M.G., Bosch T.C., Carey H.V., Domazet-Lošo T., Douglas A.E., Dubilier N., Eberl G., Fukami T., Gilbert S.F. (2013). Animals in a bacterial world, a new imperative for the life sciences. Proc. Natl. Acad. Sci. USA.

[14] Ringø E., Sperstad S., Myklebust R., Refstie S., Krogdahl Å. (2006). Characterisation of the microbiota associated with intestine of Atlantic cod (*Gadus morhua* L.): The effect of fish meal, standard soybean meal and a bioprocessed soybean meal. Aquaculture.

[15] Llewellyn M.S., Boutin S., Hoseinifar S.H., Derome N. (2014). Teleost microbiomes: The state of the art in their characterization, manipulation and importance in aquaculture and fisheries. Front. Microbiol..

[16] Wang A., Zhang Z., Ding Q., Yang Y., Bindelle J., Ran C., Zhou Z. (2021). Intestinal *Cetobacterium* and acetate modify glucose homeostasis via parasympathetic activation in zebrafish. Gut Microbes.

[17] Kanther M., Sun X., Mühlbauer M., Mackey L.C., Flynn E.J., Bagnat M., Jobin C., Rawls J.F. (2011). Microbial colonization induces dynamic temporal and spatial patterns of NF-κB activation in the zebrafish digestive tract. Gastroenterology.

[18] Yukgehnaish K., Kumar P., Sivachandran P., Marimuthu K., Arshad A., Paray B.A., Arockiaraj J. (2020). Gut microbiota metagenomics in aquaculture: Factors influencing gut microbiome and its physiological role in fish. Rev. Aquac..

[19] Ou W., Yu G., Zhang Y., Mai K. (2021). Recent progress in the understanding of the gut microbiota of marine fishes. Mar. Life Sci. Technol..

[20] Chen C.-Z., Li P., Liu L., Li Z.-H. (2022). Exploring the interactions between the gut microbiome and the shifting surrounding aquatic environment in fisheries and aquaculture: A review. Environ. Res..

[21] Kumari K., Nair S.M. (2023). Gut microbes and its physiological role in fish: Adaptive strategies for climatic variability. Outlook of Climate Change and Fish Nutrition.

[22] Zheng Q., Liu Y., Li C., Wu P., Xiao Y., Lin L., Liu Y., Zou J. (2024). Seasonal effects on bacterial community between intestine of *Collichthys lucidus* and water environment from Pearl River Estuary. South China Fish. Sci..

[23] Savard P., Fernandes T., Dao A., McMeans B., Lazar C.S. (2023). Seasons influence the native gut microbiome of lake trout *Salvelinus namaycush*. Appl. Microbiol..

[24] Ilves K.L. (2007). Molecular Systematics and Biogeography of the Holarctic Smelt Family Osmeridae (Pisces).

[25] Saruwatari T., Lopez J.A., Pietsch T.W. (1997). A revision of the osmerid genus *Hypomesus* Gill (Teleostei: Salmoniformes), with the description of a new species from the southern Kuril Islands. Species Divers..

[26] Masuda Y., Miyamoto K., Sekine S. (2023). Recirculation rate of rearing water affects growth of Japanese smelt *Hypomesus nipponensis* larvae. Fish. Sci..

[27] Xie Y., Pu X. (1984). The biological aspects of pond smelt (*Hypomesus olidus* (Pallas)) in the Shui Feng Reservoir. Acta Hydrobiol. Sin..

[28] Park J., Kim E.B. (2021). Insights into the gut and skin microbiome of freshwater fish, smelt (*Hypomesus nipponensis*). Curr. Microbiol..

[29] Ayala-Soldado N., Mora-Medina R., Molina-López A.M., Lora-Benítez A.J., Moyano-Salvago R. (2024). Evaluation of the Effectiveness of Eugenol and MS-222 as Anesthetics in Zebrafish in Repeated Exposures and Post-Anesthesia Behaviour. Animals.

[30] Jiang H., Hu J., Xie H., Zhang M., Guo C., Zhang Y., Li Y., Zhang C., Xu S., Wang D. (2023). Morphological and Molecular Functional Evidence of the Pharyngeal Sac in the Digestive Tract of Silver Pomfret, *Pampus argenteus*. Int. J. Mol. Sci..

[31] Xu L., Xiang P., Zhang B., Yang K., Liu F., Wang Z., Jin Y., Deng L., Gan W., Song Z. (2022). Host Species Influence the Gut Microbiota of Endemic Cold-Water Fish in Upper Yangtze River. Front. Microbiol..

[32] She T.W. (2020). Mechanism of Electroacupuncture at Back-Shu and Front-Mu Points of the Large Intestine Regulating Gut Microbiota in Rats with Functional Constipation. Master’s Thesis.

[33] Alves A.P., Pereira R.T., Rosa P.V. (2021). Morphology of the digestive system in carnivorous freshwater dourado *Salminus brasiliensis*. J. Fish Biol..

[34] Esfandiyari K., Pfeifer L.J., Farahani M.A., Kolbadinezhad S.M., Castro L.F., Wilson J.M. (2022). The gastric proton pump in gobiid and mudskipper fishes. Evidence of stomach loss?. Comp. Biochem. Physiol. A.

[35] De Felice E., Palladino A., Tardella F.M., Giaquinto D., Barone C., Crasto A., Scocco P. (2021). A morphological, glycohistochemical and ultrastructural study on the stomach of adult Rainbow trout *Oncorhynchus mykiss*. Eur. Zool. J..

[36] Farrag D.M.G., Azab A.M., Alabssawy A.N. (2020). Comparative study on the histochemical structures of stomach, pyloric caeca and anterior intestine in the grey mullet, *Mugil cephalus* (Linnaeus, 1758). Egypt. J. Aquat. Biol. Fish..

[37] Zhou X., Hu Z., Liu Q., Yang L., Wang Y. (2013). Feeding ecology of the non-indigenous fish *Hypomesus nipponensis* in Lake Ulungur, China: Insight into the relationship between its introduction and the collapse of the native Eurasian perch population. Mar. Freshw. Res..

[38] Bolnick D.I., Snowberg L.K., Hirsch P.E., Lauber C.L., Knight R., Caporaso J.G., Svanbäck R. (2014). Individuals’ diet diversity influences gut microbial diversity in two freshwater fish (threespine stickleback and Eurasian perch). Ecol. Lett..

[39] Tolas I., Zhou Z., Zhang Z., Teame T., Olsen R.E., Ringø E., Rønnestad I. (2025). A fishy gut feeling–current knowledge on gut microbiota in teleosts. Front. Mar. Sci..

[40] Wang L., Xue M., Yan R., Xue J., Lu Z., Wen C. (2024). Insights into chitin-degradation potential of *Shewanella khirikhana* JW44 with emphasis on characterization and function of a chitinase gene SkChi65. Microorganisms.

[41] Hirano T., Yokoyama M., Ikejima M., Shiraishi H., Hakamata W., Nishio T. (2024). Impact of chitin-derived β-N-acetyl-d-glucosaminyl-(1,4)-d-glucosamine on chitinase upregulation in *Shewanella baltica*. FEMS Microbiol. Lett..

[42] Liu G., Qiao Y., Zhang Y., Leng C., Chen H., Sun J., Fan X., Li A., Feng Z. (2020). Metabolic profiles of carbohydrates in *Streptococcus thermophilus* during pH-controlled batch fermentation. Front. Microbiol..

[43] Shah S.A.U.R., Hao Y., Tang B., Ahmad M., He D., Nabi G., Zheng J., Wan X., Wang C., Wang K. (2025). The association of seasonal dietary shift with fecal metabolome and microbiota in the captive Yangtze finless porpoise (*Neophocaena asiaeorientalis asiaeorientalis*). Environ. Res..

